# Study on the usage behavior of incidentally encountered pet health information among pet owners in the mobile media environment

**DOI:** 10.3389/fvets.2026.1867008

**Published:** 2026-07-07

**Authors:** Zixi Wang, Xinke Ren, Yifan Shu

**Affiliations:** 1 College of Veterinary Medicine, Huazhong Agricultural University, Wuhan, China; 2 Luoyang Lvzhihui Plastic Degradation Technology Co., Ltd., Luoyang, China

**Keywords:** fsQCA, mobile media, PET, pet health information, usage behavior of incidentally encountered pet health information

## Abstract

New media has become the primary channel for pet owners to acquire pet health information. Nevertheless, existing literature lacks sufficient research on pet owners’ usage behavior of incidentally encountered pet health information within the mobile media context. Focusing on exploring such usage behavior and the configurational paths of its influencing factors, this study aims to facilitate value realization of pet owners’ experiences of coming across pet health information and elevate the usage efficiency of health information resources on relevant platforms. Adopting stratified multi-stage random sampling, this research selects 362 pet owners from the main urban area of Luoyang as research respondents. Data are collected via questionnaire surveys and semi-structured interviews, and fsQCA is applied to conduct configurational analysis. The results indicate that pet owners’ usage behavior of incidentally encountered pet health information is shaped by the synergistic effect of multiple factors, and no single antecedent condition qualifies as a necessary condition. Among all antecedent variables, pet health information literacy, pet health awareness, health information characteristics, and pet health information quality serve as positive core conditions driving high levels of usage behavior toward incidentally encountered pet health information, yielding six distinct configurational paths. Internal driving factors and health information characteristics are the absent core conditions leading to non-high usage behavior. This study provides theoretical and practical references for boosting the efficiency of pet owners’ usage behavior of incidentally encountered pet health information and supporting mobile media platforms in releasing high-quality pet health information.

## 1 Introduction

The rapid development of the Internet has gradually integrated mobile media into all aspects of people’s lives. This has profoundly transformed the way people access health information. Mobile media enables users to obtain comprehensive and real-time health information, supporting more scientific decisions regarding their lifestyles and health conditions. Current research on the health information usage behavior of mobile media users mainly focuses on the communication process and application practice of human health information. It covers multiple aspects such as health information seeking (1–3), identification of false health information (4, 5), health information communication (6, 7), and the relationship between health information and health (8, 9). A common goal of these studies is to improve the efficiency of health information services for the public and optimize the paths for the public to use health information. However, most existing studies focus on individuals’ active acquisition and usage behavior of health information, with little attention paid to users’ usage behavior of health information encountered in non-active contexts.

Usage behavior of incidentally encountered health information refers to the operations such as absorption, adoption, use, comment and sharing carried out by users after accidentally obtaining health information during non-goal-oriented online activities. It possesses both the contingency and passivity of incidentally encountered information, as well as the professionalism of health information and its correlation with individual health (10). In the Internet environment, the massive and complex information ecology and big data-based precision push mechanism mean that users’ way of accessing health information is often not active retrieval, but accidental contact during the use of social media, namely incidental information. This phenomenon of incidental information provides users with a new way to obtain health information and gives rise to a unique usage behavior model (11). Mobile media users are the subjects of incidental health information usage behavior. Their internal characteristics, including personal interests, health information literacy, health information needs and health awareness, exert a critical influence on their usage behavior of incidentally encountered health information. External stimulating factors, consisting of health information quality, health information characteristics, health information types, cost perception, usefulness perception and subjective norms, also serve as critical determinants affecting mobile media users’ incidental health information usage behavior (12). Taking incidental health information as the context, Fang et al. (13) found that hypertensive patients with clear goals and a strong sense of purpose browse incidental health information in a more efficient and targeted manner. When the health information they encounter casually on social media is beneficial, they will engage in in-depth thinking about such information and further develop the willingness to share it with others. However, incidental health information may also induce anxiety. Some scholars (14) argued that health information dissemination in the new media era is fragmented and diversified. Meanwhile, the low access threshold and high openness of new media lead to the proliferation of false health information. Audiences tend to lose their judgment in the massive information environment, which easily triggers negative emotions such as anxiety and fear.

The above studies take human beings as subjects and elaborate on the theory and practice of incidental health information usage behavior. They provide solid theoretical and empirical support for this study.

In recent years, with the continuous expansion of the pet-keeping scale, the functions of pets in companionship and emotional connection have become increasingly prominent. This “quasi-kinship” emotion stimulates pet owners’ sense of “parental” responsibility and enhances their attention to pet health (15). The increasingly intimate human-pet relationship (16–18) makes people attach great importance to pet health. People obtain pet health information through channels similar to those for human health information (19). The behavior of acquiring pet health information is also profoundly influenced by the development of Internet technology. Which further deepens their usage behavior of incidentally encountered pet health information. In the mobile media environment, pet owners frequently and accidentally come into contact with various types of pet health information through algorithm recommendation and other methods, and then generate corresponding usage behaviors. However, current research in this field is still insufficient. Existing achievements mostly focus on the influencing factors and behavioral mechanisms of incidentally encountered health information (13, 20–22), while studies on pet owners’ usage behavior of incidentally encountered pet health information in the mobile media environment are relatively scarce, with basically no targeted literature results. Based on this, this study can only select some relevant achievements for demonstration, and the relevant literature review is as follows.

Studies have shown that pet owners generally use the Internet or mobile media to obtain pet health information. The content mainly covers themes such as disease prevention, behavior, and nutrition. Such information not only affects pet owners’ understanding of pet health, but also enhances their ability to communicate with veterinarians. Additionally, it plays an important role in pet health-related decisions (23). With Internet technology, some veterinarians use online tools to obtain and share animal health information. They also interact with pet owners and connect with other partners, promote the development of veterinary medicine and expand the pet medical consumption market. In addition, pet owners also use the Internet, especially social media, to search for pet health information and share their pet management experiences (24). The Internet also helps pet owners improve their pet health literacy. To make up for their lack of pet care knowledge, they often turn to the Internet for guidance. This helps them achieve precise feeding, disease prevention, and healthy behavior management. Therefore, pet information platforms should provide scientific, fast, and accurate information on pet disease prevention and health management (25). In daily life, pet owners often look for pet health information online, in addition to seeking advice from veterinarians. However, there is a risk that pet owners may obtain inaccurate or unreliable information, which could affect pet health and the relationship between veterinarians and customers (26). Kuhl et al. found through a survey that most dog owners go through an information-seeking stage before acquiring a pet. They gather resources from surveys, the internet, books, other owners, and friends. They look for information to improve the welfare of their pet dogs (27). Due to their higher Internet skills and digital literacy, younger potential owners prefer to seek information online (28).

In terms of the Internet, Kogan et al. (29) found through a study on British pet owners using the Internet to find online pet health information that the Internet (78.6%) has become the primary source of pet health information, surpassing veterinarians (72%), although the latter has higher reliability. Online pet health information helps improve pet owners’ health literacy and their understanding of veterinary advice (30). Küeper and Merle (31) further pointed out that pet owners can achieve self-education by obtaining pet health information through the Internet, thereby making more informed health decisions for sick pets and improving pet welfare. Oxley and Kogan (32) discovered that 304 rabbit keepers belonged to at least one rabbit-related Facebook group. They joined these groups to gain rabbit-raising experience. In this study, the authors demonstrated the importance of veterinary staff participating in social media groups. They noted that their involvement helps keepers obtain accurate and reliable online pet health information and seek veterinary treatment promptly. Pet owners use the Internet to find pet health information. Topics include behavior, nutrition, diet, health, and disease prevention. They regard Internet information as a supplementary resource for veterinarians. Thus, their ability to communicate with veterinarians is enhanced (33). “Internet+” is moving faster toward the “AI+” stage. AI helps pet owners access a wealth of animal health information. AI is convenient, time-saving, and offers learning opportunities. For these reasons, it has become a common tool for pet owners to seek information. However, accurate and reliable information is essential (34).

In terms of mobile media, WeChat groups have become the main platform for pet owners to spread health information. Pet owners often share their health experiences through WeChat groups or interact online with veterinarians, thereby improving the level of pet care (35, 36). Mobile social media and veterinarians together constitute the main channels for pet owners to obtain pet health information, and most pet owners are satisfied with their own understanding of pet-related health risks (37). However, a study on Iranian pet owners showed that although they are very willing to search for and use online pet health information, only half of the users care about the accuracy and credibility of online information (38). This suggests that the scientificity of online information is an important factor affecting the practicality of pet health information. The difficulty in distinguishing true from false content in the information explosion era, algorithm-aggravated homogeneous push, and fragmented presentation of information have generally plunged the public into a health cognitive dilemma (37).

The above literature provides abundant theoretical foundations and practical references for further studies on pet owners’ incidental utilization behavior of pet health information in mobile media. Nevertheless, existing studies mainly concentrate on users’ active information acquisition behaviors. Holistic investigations into the continuous behavioral chain of information encounter → health information utilization, as well as the internal correlations and synergistic mechanisms among diverse influencing factors, remain insufficient. With the rapid advancement of digital technologies and the widespread popularization of mobile media, profound changes have taken place in public lifestyles. Excessive reliance on mobile media has gradually become a universal social norm. Accordingly, utilization behaviors generated by pet owners’ unintentional exposure to pet health information or passive continuous browsing of relevant content have become vital research topics. In addition, the interactive effects among antecedent variables on incidental pet health information utilization behaviors, together with the influence intensity of each factor, are regarded as core research priorities.

On this basis, pet health information in the mobile media is taken as the research object. The utilization behaviors and configurational influence paths of pet owners’ incidental exposure to pet health information are systematically analyzed, and key factor configurations driving such behaviors are identified. This study helps expand the application scope of information encounter theory, and clarifies behavioral characteristics and driving determinants of pet owners in the process of incidental pet health information acquisition. Furthermore, the results offer empirical evidence for media platforms to optimize pet health information recommendation strategies and improve the communication quality of pet health information. Meanwhile, valuable references are provided for pet health publicity institutions to formulate targeted pet health education programs.

## 2 Materials and methods

### 2.1 Research instruments

The Scale for Usage Behavior of Incidentally Encountered Pet Health Information was adapted from the scale developed by Tian et al. (10) and further modified to fit the specific objectives of the present study (Table 1). The scale content was carefully revised and optimized in accordance with the research framework to ensure clarity and conciseness. The scale underwent two rounds of modifications by four pet owners with master’s degrees, and five additional pet owners provided feedback after completing the scale, resulting in high content validity. The scale consisted of 8 dimensions and 18 sub-factors, specifically including pet health information literacy (three sub-factors), pet health awareness (two sub-factors), pet health information needs (two sub-factors), subjective norms (two sub-factors), perceived cost of pet health information (two sub-factors), pet health information characteristics (two sub-factors), pet health information quality (two sub-factors), and usage behavior of incidentally encountered pet health information (three sub-factors).

**Table 1:** Scale for usage behavior of incidentally encountered pet health information.

Category	Code	Dimension	Symbol	Factor
Internal drivers	a11	Pet health information literacy	v1	I can distinguish the scientificity of pet health information.
			v2	I have the ability to search for pet health information.
			v3	I have relatively rich knowledge and experience in pet health.
	a22	Pet health awareness	v4	I believe pet health information is important for promoting pet health.
			v5	I attach importance to my pet’s health status.
	a33	Pet health information needs	v6	I have needs for pet health information.
			v7	My family members or pet-keeping friends have needs for pet health information.
External stimuli	a44	Subjective norms	v8	Others’ evaluations and attitudes towards incidentally encountered pet health information will affect my subsequent behaviors.
			v9	Attitudes of people around me or the social environment towards incidentally encountered pet health information will affect my subsequent behaviors.
	a55	Perceived cost of pet health information	v10	From an economic perspective, the incidentally encountered pet health information is feasible to implement.
			v11	From a time perspective, the incidentally encountered pet health information is feasible to implement.
	a66	Pet Health information characteristics	v12	I believe the incidentally encountered pet health information is easy to understand.
			v13	I believe the incidentally encountered pet health information has practical utility.
	a77	Pet health information quality	v14	I believe the incidentally encountered pet health information is scientific and accurate.
			v15	I believe the source of the incidentally encountered pet health information is reliable.
Usage behavior of incidentally encountered pet health information	a88		v16	On the basis of understanding, I will share, discuss, forward, and seek solutions for the incidentally encountered pet health information with others.
			v17	On the basis of understanding, I will reserve the incidentally encountered pet health information as relevant knowledge for communication with veterinarians.
			v18	On the basis of understanding, I will practice the incidentally encountered pet health information (e.g., prevention, health decision-making, inspection, recalling experience, etc.).

In the first revision round, the eight dimensions were consolidated into three clusters: internal drivers, external stimuli, and usage behavior of incidentally encountered pet health information. The 4 pet owners with master’s degrees discussed the scientificity of the scale and revised the expressions of 10 sub-factors to improve comprehensibility. In the second round, five pet owners were invited to complete the scale in a pilot test. Based on their feedback, wording of four sub-factors was refined. The scientific quality of the scale was re-assessed by the four postgraduate pet owners. The formal scale achieved a Cronbach’s alpha coefficient of 0.826, indicating good reliability. Principal component analysis was performed to examine factor validity. The eight dimensions collectively explained 74.695% of the total variance, with factor loadings ranging from 0.769 to 0.876, indicating good factor structure validity. A 5-point Likert scale was adopted for all items, ranging from strongly inconsistent (1) to strongly consistent (5). Higher scores represented a higher level of the corresponding construct.

### 2.2 Study design

Pet owners in Luoyang City were selected as the participants. The inclusion criteria were as follows: (1) having kept a dog or cat for no less than 2 months with relevant care experience; (2) being active mobile media users who regularly browse and use domestic and foreign websites, Tik Tok, Kuaishou, WeChat, QQ, Weibo, and other media platforms, with experiences of encountering and utilizing pet health information in daily media use.

**Figure 1:**
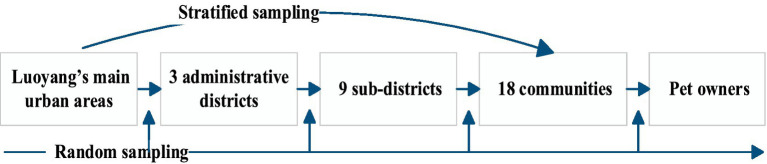
Stratified multi-stage random sampling.

This study was supported by the research project of Luoyang Lvzhihui Plastic Degradation Technology Co., Ltd., and the survey scope was limited to the main urban areas of Luoyang. This study was carried out in two stages. Questionnaires and interviews were implemented from August 4 to 24, 2025, while data supplementation and thesis writing were completed from January 26 to February 28, 2026. As a complete list of local pet owners could not be obtained individually, random sampling targeting all pet raisers across communities in Luoyang was impractical. Accordingly, stratified multi-stage random sampling was adopted to collect samples in accordance with research objectives, with no fewer than 400 pet owners planned to be investigated. Based on the administrative affiliation of Luoyang’s main urban areas, stratified multi-stage random sampling was applied to determine survey regions at three levels: main urban districts, sub-districts and communities. Random sampling was further conducted to select pet owners within each community, data collection was conducted through offline field investigations. The detailed sampling procedures were as follows. First, three administrative districts, namely Luolong District, Jianxi District and Xigong District, were randomly selected from five main urban districts of Luoyang. Second, three sub-districts were randomly chosen from each selected district, totaling nine sub-districts. Subsequently, two communities were extracted at random from each sub-district, forming a total of eighteen communities. Finally, with assistance from community staff, lists of dog owners in each community were summarized. Twenty-five respondents were randomly selected from every community, amounting to 450 participants in total (Figure 1). Questionnaires were adopted for data collection. After contacting respondents, they were explained survey contents and relevant specifications. Eventually, 388 individuals signed informed consent forms and took part in the investigation, while 62 others failed to meet inclusion criteria or declined participation. Questionnaires were distributed and retrieved on-site. A total of 388 questionnaires were issued and 375 were recovered (owing to unexpected urgent affairs, this questionnaire will not be submitted henceforth). After eliminating incomplete responses, 362 valid questionnaires were obtained (Table 2), the effective response rate was 96.53%, and the demographic characteristics of pet owners are presented in Table 2:male (129), female (233); ≤60 years old (39), 61–70 years old (193), ≥71 years old (111); primary school or below (7), junior high school/senior high school/technical secondary school (104), junior college or above (251); living alone (74), not living alone (288).

**Table 2:** Demographic characteristics.

Demographic characteristics	Category	Number	%
Gender	Male	129	36%
	Female	233	64%
Age	≤60 years old	58	16%
	61–70 years old	193	53%
	≥71 years old	111	31%
Educational level	Primary school or below	7	2%
	Junior high school/senior high school/technical secondary school	104	29%
	Junior college or above	251	69%
Residential status	Living alone	74	20%
	Not living alone	288	80%

### 2.3 Research methods and data processing

The study adopted the literature review method to collect relevant literature and sort out the current state of existing research. Questionnaire surveys and interviews were collected through offline field investigations. Descriptive and exploratory analyses of the data were performed using SPSS 26, fsQCA is then adopted for configurational research, and research results were discussed in combination with semi-structured interviews. This study is a mixed-methods study.

fsQCA was proposed by American scholar Professor Charles C. Ragin in 1987. It is a case-oriented method that can analyze both quantitative and qualitative data. By using antecedent conditions, it explores the conditional combinations that lead to outcomes. Different conditional combinations may result in the same outcome pathways, with greater emphasis on the diversity of causal relationships. Meanwhile, causal effects are not consistent (40). Therefore, through the continuous integration of empirical data and relevant theories, the study constructed the causal relationship of the research topic from the sample data.

To ensure the stability and reliability of the analysis, the number of antecedent conditions in fsQCA research generally does not exceed 7–8. The number of cases is suitable for small to medium-sized cases, which are generally actual case data (e.g., enterprises, typical cases, units, etc.). However, there is no requirement for individual survey data (41). In general, a sufficient sample size can ensure the generation of enough combination patterns during the analysis, making the analysis results more representative and reliable (e.g., scholars such as Du conducted (42) research using 2,622 questionnaire data, Yu conducted (43) research using 335 questionnaire data, and Wu conducted (44) research using468 questionnaire data).

Primary data were collected via semi-structured interviews. To gain an in-depth understanding of pet owners’ usage behavior of incidentally encountered pet health information and provide respondents with more opportunities to express their views and feelings, a semi-structured interview method was used in this study for supplementary data collection. An interview outline was designed in accordance with the research objectives to obtain more authentic and in-depth responses.

The interview outline was framed based on the scale measuring usage behavior of incidentally encountered pet health information. The interview content covered seven dimensions, namely pet health information literacy, pet health awareness, pet health information demand, subjective norms, perceived cost of pet health information, pet health information characteristics, pet health information quality, and usage behavior of incidentally encountered pet health information. During the interviews, researchers followed the outline while making flexible adjustments according to on-site conditions. Specifically, the sequence, form and wording of questions could be modified as appropriate, and valuable topics were probed in depth to fully grasp the perspectives of pet owners.

In terms of sampling, random sampling was adopted from valid questionnaire respondents to avoid respondent fatigue and homogeneous data caused by extensive interviews. Specific procedures were as follows: all 362 pet owners across 18 communities were assigned serial numbers. One respondent was randomly selected from each community, with an additional respondent drawn from communities containing 20 or more valid samples. A total of 29 pet owners were recruited, yet one withdrew during the interview process, leaving 28 valid interviewees. To protect respondents’ privacy, serial codes A1 to A28 were used in place of real names. Researchers respected all verbal and nonverbal expressions of pet owners without any inducement or intervention. Hard-copy notes and audio recordings were collected simultaneously during interviews, with each interview limited to 30 min.

Within 12 h after each interview, researchers transcribed all interview data into textual transcripts with reference to audio recordings where necessary. Integrated and revised transcripts constituted the thematic research dataset. Interview excerpts covering identical themes were categorized as the same data type, and representative interview narratives from respondents were selected as research cases.

## 3 Results

### 3.1 Data calibration

Data calibration constitutes a critical procedure in fsQCA. Its purpose is to transform raw data into qualified fuzzy-set data so that clear set membership can be assigned to all cases in subsequent analysis. Two main approaches are available for fuzzy-set calibration, namely the direct method and the indirect method. The direct calibration method establishes three qualitative anchors based on theoretical and practical grounds: full membership, the crossover point, and full non-membership, with calibration implemented via the algorithm provided by software. The indirect calibration method requires researchers to assign multiple values between 0 and 1 to each condition based on their own judgment. In this study, the direct calibration method was adopted, following the work of Jin et al. (45) and considering the characteristics of the 5-point Likert scale. In accordance with the actual sample distribution, values of 5, 3.5, and 1 were set as calibration anchors representing full membership, the crossover point, and full non-membership, respectively. All variables were calibrated using fsQCA 4.1 software, and original scores were converted into membership scores ranging from 0 to 1. These membership scores indicate the degree to which each case belongs to the outcome set. For cases where the fuzzy-set membership score was exactly 0.5 after calibration, the value was adjusted to 0.499 with reference to Adrian and Du (46) to prevent such cases from being excluded in later analysis.

### 3.2 Necessity test of single conditions

Consistency serves as a key indicator for judging whether a single conditional variable forms a necessary or sufficient condition for the outcome variable and allows rapid screening of necessary conditions with extremely high consistency. A threshold value of 0.9 is commonly used for identifying necessary conditions. A condition with a consistency score higher than 0.9 can be regarded as a necessary condition for the outcome variable; otherwise, it is not considered a necessary condition (47). As shown in Table 3, the consistency scores of all antecedent variables are below 0.9. This demonstrates that no single factor constitutes a necessary condition for the usage behavior of incidentally encountered pet health information, and each antecedent variable provides a certain degree of explanatory power for the outcome.

**Table 3:** Analysis of necessary conditions.

Conditional variable	High usage behavior of incidentally encountered pet health information		Non-high usage behavior of incidentally encountered pet health information	
	Consistency	Coverage	Consistency	Coverage
a11	0.764	0.793	0.746	0.459
~a11	0.479	0.761	0.663	0.625
a22	0.761	0.802	0.758	0.474
~a22	0.500	0.777	0.683	0.630
a33	0.778	0.783	0.773	0.462
~a33	0.465	0.776	0.637	0.630
a44	0.672	0.843	0.652	0.484
~a44	0.589	0.740	0.788	0.588
a55	0.684	0.833	0.687	0.497
~a55	0.587	0.760	0.769	0.591
a66	0.777	0.824	0.729	0.458
~a66	0.489	0.752	0.721	0.658
a77	0.800	0.854	0.680	0.430
~a77	0.466	0.710	0.769	0.695

~ denotes negation in set operations, i.e., the absence of the condition.

### 3.3 Configurational analysis of conditions for high and non-high usage behavior of incidentally encountered pet health information

Configurational analysis was conducted using fsQCA 4.1 software to identify the causal paths leading to high usage behavior and non-high usage behavior of incidentally encountered pet health information. According to Ragin and Fiss (48) and the characteristics of the sample data, the frequency threshold for high usage behavior was set at 6 (accounting for approximately 1.5% of the total sample). The raw coverage threshold was set to 0.8, and the PRI consistency threshold was set to 0.8 (49). For the analysis of non-high usage behavior, since the initial PRI values were all below 0.70, the PRI consistency threshold was lowered to 0.60 in line with Mo and Jiang (50). It should be noted that configurations may show significant inconsistency when PRI values are excessively low; therefore, the analysis of non-high behavior is regarded as exploratory and for reference only.

The fsQCA results are presented in Table 4. Six configurations (W1–W6) were identified for high usage behavior of incidentally encountered pet health information, and one configuration (Y1) was identified for non-high usage behavior. Three solutions, namely the complex solution, parsimonious solution, and intermediate solution, were generated through fsQCA 4.1. Following commonly adopted interpretation approaches in previous studies, a combined analysis of the intermediate solution and parsimonious solution was performed. Antecedent conditions appearing in both the parsimonious solution and intermediate solution were defined as core conditions for the outcome, whereas those appearing only in the intermediate solution were classified as peripheral conditions.

**Table 4:** Configurational analysis of factors influencing usage behavior of incidentally encountered pet health information.

Conditional variable	High usage behavior of incidentally encountered pet health information						Non-high usage behavior of incidentally encountered pet health information
	W1	W2	W3	W4	W5	W6	Y1
a11	⦁	⦁	●	⦁	⦁	⦁	⊗
a22	-	⦁	●	⦁	⦁	o	⊗
a33	⦁	⦁	⊗	⦁	-	⦁	⊗
a44	o	–	o	o	⦁	⦁	o
a55	–	–	o	o	⦁	o	o
a66	⦁	⦁	●	–	o	o	⊗
a77	●	●	–	●	●	●	o
Consistency	0.942	0.937	0.964	0.947	0.966	0.977	0.817
Raw coverage	0.387	0.475	0.243	0.329	0.283	0.217	0.331
Unique coverage	0.017	0.075	0.024	0.009	0.014	0.004	0.331
Solution consistency	0.928						0.817
Solution coverage	0.563						0.331

“●” indicates the presence of a core condition; “⦁” indicates the presence of a peripheral condition; “⊗” indicates the absence of a core condition; “o” indicates the absence of a peripheral condition; “–” indicates that the condition is irrelevant to the outcome.

The results show that among the six configurations for high usage behavior of incidentally encountered pet health information (W1–W6), all configurations achieve consistency values above 0.9, indicating high validity. Specifically, the raw coverage of each configuration exceeds 0.21, suggesting that each configuration can explain more than 21% of the cases with high usage behavior of incidentally encountered pet health information. The unique coverage of cases that can be explained by only a single path ranges from 0.4 to 7.5%. The overall solution coverage is 0.563, meaning that the identified configurations account for 56.3% of cases with high usage behavior of incidentally encountered pet health information, supporting the reliability of the findings.

In-depth analysis of the antecedent configuration for non-high usage behavior of incidentally encountered pet health information (Y1) reveals that this path consists of the absence of four core conditions and the absence of three peripheral conditions, with a configuration consistency of 0.817 (>0.75). The overall solution coverage is 0.331, indicating that this configuration explains 33.1% of cases with non-high usage behavior of incidentally encountered pet health information. As the PRI consistency threshold is only 0.618, this result is regarded as exploratory and provides reference for understanding the formation of non-high usage behavior of incidentally encountered pet health information.

### 3.4 Robustness test

Following mainstream practices in QCA research, a robustness test was conducted on the configurational pathways of usage behavior of incidentally encountered pet health information by adjusting the frequency threshold to 5 and the consistency threshold to 0.90. The six newly generated configurational pathways (W1–W6) were consistent with the original configurations in terms of core conditions, with only minor differences in peripheral conditions and slight changes in the sequence of partial pathways. The solution consistency of the revised model was 0.916, indicating that 91.6% of cases matching the six configuration patterns exhibited the targeted usage behavior, and the solution coverage reached 0.601, demonstrating that the identified configurations could explain 60.1% of outcome cases. No changes were detected in the configuration of non-high usage behavior of incidentally encountered pet health information (Y1), which verifies the robustness of the research findings.

## 4 Discussion

### 4.1 Analysis of high usage behavior of incidentally encountered pet health information

According to the core conditions of the configurations, the six paths can be classified into three types: the information quality-internal driver synergy type, the subject quality-information feature driven type, and the information quality-external driver synergy type. The information quality-internal driver synergy type (W1, W2, W4, W6) takes high pet health information quality as the core condition, with some high internal drivers and a small number of external stimuli as peripheral conditions, which jointly generate high usage behavior of incidentally encountered pet health information. The subject quality-information feature driven type (W3) takes pet owners’ high subject quality (high pet health information literacy and high pet health awareness), non-high pet health information needs and high pet health information characteristics as core conditions, and some non-high external stimuli as peripheral conditions, which together lead to high usage behavior of incidentally encountered pet health information. The information quality-external driver synergy type (W5) regards high pet information quality as the core condition, supplemented by other peripheral factors to jointly stimulate the targeted usage behavior. Each configurational pathway is analyzed in detail as follows.

#### 4.1.1 Information quality-internal driver synergy type

The configuration paths of this type are all characterized by high pet information quality as the sole core condition, and a relatively high proportion of internal drivers among peripheral conditions. This indicates that when pet owners encounter high-quality pet health information in mobile media and possess corresponding health beliefs (such as information literacy or information needs), their usage behaviors including information absorption, adoption and sharing can be effectively triggered under partial external stimulation. A generation mechanism dominated by information quality and coordinated by internal drivers is thus formed, highlighting the vital role of pet health information quality. Usage behavior can still be triggered even when internal drivers are not positioned as core conditions, representing a group of pet owners highly sensitive to information quality.

Configuration W1. When high pet health information quality is taken as the core condition, supplemented by high pet health information literacy, high pet health information needs, non-high subjective norms and high pet health information characteristics as peripheral conditions, high usage behavior of incidentally encountered pet health information can be generated. Pet health awareness and perceived cost of pet health information exert no influence on the outcome. This shows that when pet owners encounter high-quality health information in mobile media, if they have relatively high information literacy and needs, and the presentation of pet health information is distinct, positive usage behavior of incidentally encountered pet health information will occur even with a low level of external subjective norms. Some interview results confirm this path. Respondent A12 has raised a dog for 1 year and joined three WeChat groups to monitor the health status of his pet dog. He often encounters dog-raising experiences forwarded by netizens in the groups, which has cultivated his basic pet health literacy. Among massive incidentally encountered information, he understands and memorizes high-quality and practical content as nursing knowledge for his dog and references for future veterinary consultations. Respondent A11 has raised a dog for 3 years with rich breeding experience, who attaches great importance to pet health and holds explicit demands for professional pet health knowledge. She stated that “for health information of dogs encountered in mobile media, I will conduct multi-party judgment, and finally only share and memorize reliable information.” This originated from a skin disease suffered by her Pomeranian 2 years ago. “At that time, I consulted netizens through WeChat groups and implemented the nursing knowledge shared without strict identification. As a result, the veterinary drugs purchased were inconsistent with the disease, which made family members busy for an extra week.” This experience made her highly vigilant about information quality. The above interview evidence verifies the promoting effect of information characteristics, information literacy and information needs on usage behavior under the core premise of information quality.

Configuration W2. High pet health information quality was identified as the core condition. High pet health information literacy, high pet health awareness, high pet health information needs, and high pet health information characteristics were included as peripheral conditions. Such a combination can generate high usage behavior of incidentally encountered pet health information. Subjective norms and perceived cost of pet health information showed no effect on the outcome. This configuration indicates that under the stimulation of high-quality pet health information, pet owners with complete internal drivers (literacy, awareness, and needs) and the ability to recognize distinct information features tend to exhibit positive usage behavior. This pattern holds regardless of subjective norms or perceived cost of pet health information. Supporting evidence was obtained from interviews. Respondent A5, with two and a half years of dog-raising experience, self-assesses her pet health literacy at a relatively high level and enjoys disseminating pet health knowledge through WeChat groups, and frequently encounters pet health information via mobile media. She stated that information on mobile platforms is abundant but not always reliable. She emphasized that pet owners should value the health of their “fur babies,” accumulate scientific and effective knowledge, and memorize such information to improve pet welfare. This case confirms that pet owners with sufficient internal driving factors and the ability to identify distinctive information features are more inclined to utilize incidentally encountered pet health information under the drive of high-quality information.

Configuration W4. High pet health information quality was recognized as the core condition. High pet health information literacy, high pet health awareness, high pet health information needs, non-high subjective norms, and non-high perceived cost of pet health information were used as peripheral conditions. This combination can generate high usage behavior of incidentally encountered pet health information. Pet health information characteristics showed no significant effect. A slightly different mechanism was observed. As long as pet health information is scientific, effective, and reliable, pet owners with complete internal drivers are likely to show positive usage behavior. This occurs even under conditions of low subjective norms and low perceived cost, regardless of the practical value of the information itself. The experience of Respondent A8 provides evidence. With 5 years of cat-raising experience, she has developed rich nursing knowledge. During the COVID-19 pandemic, she remained unaffected by widespread anxiety over zoonotic diseases in WeChat groups. She actively selected authoritative information and applied it to the health monitoring of her cat, which enhanced her confidence in pet care. This behavior reflects that when pet owners encounter high-quality pet health information, positive synergy among the three internal drivers enables them to demonstrate high usage behavior of incidentally encountered information even under external environments characterized by low subjective norms and low perceived cost.

Configuration W6. High pet health information quality was taken as the core condition. High pet health information literacy, non-high pet health awareness, high pet health information needs, high subjective norms, non-high perceived cost of pet health information, and non-high pet health information characteristics were used as peripheral conditions. This combination could generate high usage behavior of incidentally encountered pet health information. This configuration covered all antecedent conditions, showing a complex synergistic mechanism among various factors. When pet owners encountered high-quality health knowledge, coupled with high information literacy and needs, external subjective norms could replace non-high health awareness to drive usage behavior. This theoretical logic has been verified in prior research, in which external evaluations and attitudes can make up for deficiencies in internal awareness (51). The behavior of Respondent A17 was consistent with this mechanism. As both a dog owner and a veterinarian, mobile media was her main channel for communicating with clients and conducting business. When encountering pet health information, she paid more attention to content with high likes or social popularity. She then posted professional opinions based on her expertise on Tik Tok and WeChat groups. Her professional identity as a veterinarian endowed her with strong subjective norms, information literacy, and information needs. Information quality supported her in transforming incidentally encountered content into more rigorous and scientific professional outputs. This case verified the functional logic of internal-external synergy and norm compensation. Respondent A1 has nearly 3 years of dog-raising experience, and she holds the view that public opinions can stimulate her beliefs regarding pet health protection.

The above paths collectively indicate that the scientificity and reliability of pet health information itself are the key factors triggering usage behavior. They can coordinate with pet owners’ personal literacy, needs and partial external stimuli to jointly promote information utilization. On this basis, W1 shows a promotion mechanism dominated by information literacy and needs with distinct information characteristics; W2 presents strong synergy of the three internal drivers; W4 highlights the external environmental advantage of high pet health information quality on the basis of complete and intensive internal factor coordination; W6 reveals the unique mechanism of subjective norms replacing pet health awareness. Therefore, it is recommended that mobile media platforms and animal-related accounts strengthen the quality review of pet health information and label scientifically accurate information. Meanwhile, the reporting mechanism for false pet health information should be optimized to facilitate the dissemination of high-quality pet health information. Pet owners may also actively improve their own information literacy to avoid missing high-quality pet health information due to the absence of internal driving factors.

Based on the analysis of high usage behavior of incidentally encountered pet health information, relevant literature provides direct support for this study. A study on pet owners’ use of social media to discuss dog health showed that mobile media, as a platform for pet owners to exchange pet health information, can effectively improve dog welfare (52). On the popular mobile media Tik Tok, half of the dog health videos focus on scientifically verified health knowledge, while the other half focus on practical content. Videos released by veterinarians are more reliable (53), which also confirms the core role of information characteristics and information quality in driving usage behavior in this study. Many scholars have found that owners who care about their dogs’ health usually actively collect more information to help understanding before seeking veterinary diagnosis (54), which is consistent with the information quality-internal driver synergy type. When pet owners have a certain ability to identify information, the stimulation of authoritative and high-quality information will directly trigger their information-seeking behavior.

In terms of factors influencing health information usage behavior, studies on human health information usage behavior provide relevant evidence for this research. A study on Little Red Book users found that the sharing behavior of incidentally encountered information is affected by three dimensions: user factors, environmental factors, and information factors. Users’ information literacy, social environment, information usefulness, and credibility can all effectively trigger usage behavior (55). Research by Fallatah et al. (56) also confirmed that health information quality and presentation, willingness to help others, and demand for in-depth understanding of information can all promote users to share incidentally encountered health information, while negation of information will prompt users to ignore it. The above studies all indicate that high-level user subject quality, social norms, information characteristics, and information quality have a promoting effect on usage behavior, which is basically consistent with the conclusions of this study.

#### 4.1.2 Subject quality-information feature driven type

This type of configuration path takes pet owners’ subject quality (information literacy and awareness) and health information characteristics as positive core conditions, showing a dual-track driving feature. It indicates that when some pet owners have high information literacy and health awareness, and the characteristics of incidentally encountered pet health information are sufficiently distinct, usage behavior can still be effectively triggered even if other internal and external conditions are absent. A generation mechanism jointly driven by subject quality and information characteristics is thus formed. This suggests that when encountering pet health information, pet owners’ usage behavior is closely related to their own characteristics, reflecting differences among different pet owner groups.

Configuration W3 points out that high usage behavior of pet health information can be generated when high pet health information literacy, high pet health awareness, and non-high pet health information needs serve as core conditions, with non-high subjective norms and non-high perceived cost of pet health information as peripheral conditions. However, pet health information quality has no impact on the outcome. This presents a new path: when pet owners have high information literacy and health awareness but lack clear information needs, usage behavior can be triggered in an external environment with low subjective norms and low perceived cost of pet health information, as long as the characteristics of the incidentally encountered information are sufficiently distinct. Some respondents fit this type. Respondent A28, with 6 years of cat-raising experience, self-assesses rich experience in cat breeding. She considers pet hospital fees high and thus pays extra attention to daily health prevention. When encountering pet health information in mobile media, she selects and saves content that is simple and easy to understand, using it as a basis for future prevention and health checks of her cat. For information that is difficult to understand and judge, she tends to skip it directly to avoid untested information interfering with her knowledge reserve. In cases of deteriorating pet health, she ignores irrelevant pet health information and chooses to consult veterinarians for professional diagnosis. Respondent A6, with 4 years of experience raising both cats and dogs, often browses web pages and chats on social media via mobile phones to pass the time. He believes he has strong pet health awareness and literacy. For incidentally encountered pet health information that is easy to understand and simple to operate, he occasionally checks his pets subjectively for similar diseases on the basis of understanding. This confirms that when pet owners have sufficient subject quality, the presentation characteristics of information can replace content authority and become the dominant force driving the usage behavior of incidentally encountered information.

The above paths illustrate that there are some pet owner groups who are sensitive to information characteristics. Due to their ability to identify information quality, they tend to digest information that is easy to understand and practically useful. This finding expands the understanding of users’ usage behavior of incidentally encountered pet health information and presents a sensitive usage behavior model. Therefore, it is recommended that animal-related accounts on mobile media improve the readability of pet health information and make the knowledge presentation form more distinct on the basis of ensuring information effectiveness. Pet owners are advised to read more professional literature to enhance their reading and judgment abilities.

Relevant literature provides direct evidence for this study, Surveys have shown that 80% of veterinarians approve of clients searching for pet health information online. However, more than half of veterinarians believe that clients do not truly understand the information obtained, and this phenomenon may have a negative impact on the veterinarian-client relationship (57), highlighting the core status of pet owners’ health literacy. Varmazyar et al. (53) made similar findings: owners of non-brachycephalic dogs usually have higher health awareness and tend to discuss life-related topics such as alternative and complementary therapies and diet training with peers through social media, actively trying to reduce potential health problems of their dogs. This behavior pattern confirms the path of the subject quality-information feature driven type.

#### 4.1.3 Information quality-external driving synergy type

This type of configuration takes high pet health information quality as the core driving condition, accompanied by abundant external factors serving as peripheral conditions. This indicates that when some pet owners encounter high-quality pet health information on mobile media, they can generate usage behavior through the coordination of partial internal factors under the effects of external elements such as information cost. It demonstrates the diversity of usage behavior among pet owners who are highly sensitive to information quality.

Configuration W5 shows that high usage behavior of pet health information can be generated when high pet health information literacy and high pet health information quality serve as core conditions. High pet health awareness, high subjective norms, high perceived cost of pet health information, and non-high pet health information characteristics are included as peripheral conditions. Pet health information needs exert no effect on the outcome. This suggests that when pet owners possess high information literacy, usage behavior can be triggered as long as the quality of incidentally encountered information is sufficiently high. This pattern holds regardless of information needs, even with non-high information characteristics, and is guided by personal awareness, external norms, and perceived cost. This path is supported by the following interview evidence. Respondent A23, with 7 months of dog-raising experience, actively follows pet health information encountered in mobile media. He is capable of understanding professional terms and making judgments on encountered pet health information. He noted that high-quality information that triggers online debates often attracts his participation, stating that “knowledge from online debates improves the pet-raising experience of dog owners.” He also shares debate outcomes with other dog owners. Respondent A2, with 9 years of dog-raising experience, regards his dog as family and attaches great importance to daily health. When encountering interesting pet health information in WeChat groups or web pages, he first conducts self-evaluation regardless of presentation format. After confirming its feasibility, he shares the information with fellow pet owners. He holds that the frequency of paying attention to and sharing incidentally encountered pet health information is correlated with external public opinion environments, and he tends to share and store convenient and preventive health knowledge more frequently. The above two cases interpret one dimension of pet owners’ usage behavior toward incidentally encountered pet health information.

The above paths demonstrate that pet owners who are more sensitive to information quality can sharply identify high-quality pet health information in mobile media through their high information literacy. Usage behavior is triggered by the synergy of internal and external factors, showing an authority-sensitive usage pattern. Therefore, it is recommended that popular science accounts on mobile media attach importance to the practicality and popularity of content, and provide users with identifiable and verifiable quality labels. Mobile media platforms may also introduce authoritative source marking to form a benign communication chain from authority identification to community diffusion. Pet owners may actively improve their pet health information literacy to further strengthen their ability to judge information quality. In this way, high-quality incidentally encountered pet health information can be transformed into effective resources for daily health management.

Social norms can raise the frequency with which pet owners quickly search for pet health information online. Nevertheless, evaluations of pet health information conducted by veterinarians reveal disparities in the accuracy and completeness of information across different websites (58). Similarly, Hofmeister et al. (39) found that pet owners obtain pet health information via multiple channels including the Internet, veterinarians, family and friends, magazines, television and newspapers; meanwhile, the accessibility and credibility of pet health information serve as critical determinants of pet owners’ willingness to consult veterinarians. When summarizing studies on Internet health information, many scholars pointed out that core members of online communities for diabetes patients often have more disease experience, can accurately identify practical and feasible health information needed by other users, and thus provide abundant reliable information resources for peers. This phenomenon also exists among cancer patients (59), which highlights the promotional effect of community demands on the adoption and sharing of high-quality health information, and is highly consistent with the path logic of the information quality-external driver synergy type.

### 4.2 Analysis of non-high usage behavior of incidentally encountered pet health information

As an exploratory configuration path, Y1 indicates that non-high usage behavior of incidentally encountered pet health information can be effectively triggered when pet health information literacy, pet health awareness, pet health information needs, and pet health information characteristics serve as core absent conditions, and subjective norms, perceived cost of pet health information, and pet health information quality act as peripheral absent conditions; this configuration belongs to the type dominated by internal driver absence and coordinated by external stimulus inhibition. This demonstrates that the absence of the three internal drivers is the core factor inhibiting pet owners’ usage behavior of incidentally encountered pet health information. When pet owners lack subject quality and information needs, and the incidentally encountered pet health information fails to attract their attention with distinct characteristics, they will ignore such information in the absence of other external stimuli. Similar cases were found in interviews. Respondent A14, with 6 years of dog-raising experience, stated that he had little motivation to obtain pet health information. He was busy with business affairs daily and did not regard pet health as an important matter. Therefore, he rarely paid attention to pet health information encountered in social circles or media. This case illustrates the importance of high internal drivers and external stimuli. Conversely, when internal drivers and external stimuli are all absent or non-high, they will jointly generate non-high usage behavior of incidentally encountered pet health information.

Relevant studies have also verified that pet owners’ levels of e-health literacy lead to varying degrees of recognition of prescription information provided by veterinarians (60). Additionally, if owners fail to pay attention to their dogs’ mental health, the dogs are prone to developing progressive psychological distress (61).

### 4.3 Critical perspectives

This study clarifies the relative importance of the seven antecedent configurational factors through an analysis of high and non-high usage behavior of incidentally encountered pet health information. It is worth noting that some research results are different from this study. Tian et al. (11) found in a study on older adults using social media that information quality, information relevance, and health information needs have a significant impact on the usage behavior of incidentally encountered health information. Other studies have found that usage context has a positive impact on health information encountering behavior by influencing emotional states, while the positive impact of information characteristics on usage behavior has not been verified (62). Some influencing factors pointed out in the above studies are different from those in this study. For instance, information needs failed to exert positive core effects across all seven configurations, whereas information characteristics served as a positive core condition in one configuration. This difference may stem from the different subjects. People may have different attention patterns and behavioral logics towards their own health information and pet health information. In addition, pet owners may have different usage behaviors due to different types of pets raised. Many scholars have also found that when providing health advice, owners of non-brachycephalic dogs perform better, while owners of brachycephalic dogs are passive and show negative responses to positive external information popularization (53). This suggests that factors such as pet type and raising experience may regulate pet owners’ information behavior patterns, which is worthy of further exploration in follow-up studies.

## 5 Research limitations and insights

This study has several limitations. First, regarding variable selection, seven antecedent conditions were chosen from the dimensions of internal and external factors, which is supported by sufficient theoretical and practical evidence and meets the basic requirements of QCA for condition selection. Nevertheless, some potential variables and path combinations remain to be further explored. Second, the usage behavior of incidentally encountered pet health information is formed through the coordination of multiple factors, and the interaction mechanism among these factors requires further analysis. Future research may adopt more qualitative methods or longitudinal data for in-depth investigation. Finally, the sample of this study was mainly collected from Luoyang City. The findings carry certain regional characteristics. Future studies can be extended to a wider geographical range and include more diverse groups of pet owners and antecedent conditions to enhance the generalizability of the conclusions.

## 6 Conclusion and recommendations

No single conditional variable can act as a necessary condition affecting pet owners’ usage behavior of incidentally encountered pet health information, and such behavior is shaped by the synergistic effects of multiple factors. The innovation of this study lies in its adoption of a mixed-methods research approach. It reveals that pet owners’ usage behavior of incidentally encountered pet health information within unplanned, unexpected information exposure scenarios is driven by seven configurational paths, and distinguishes core and peripheral conditional factors within these paths. This study elaborates on how the interaction of diverse factors motivates pet owners to use incidentally encountered pet health information and generates positive outcomes of such usage behavior. It delivers novel theoretical perspectives and methodological support for advancing pet health research. Configurations corresponding to high usage behavior of incidentally encountered pet health information fall into three categories: the information quality-internal driver synergy type, the subject literacy-information feature driven type, and the information quality-external driver synergy type. All three types feature combined configurations of internal driver and external stimulus conditional variables. Pet health information quality functions as a positive core condition of paramount importance across all three configuration types. Configurations representing non-high usage behavior of incidentally encountered pet health information reflect an inhibitory mechanism marked by the absence of the three core internal driver factors, alongside missing external stimulus synergy or non-high peripheral conditions, which are classified as the type dominated by internal driver absence and coordinated by external stimulus inhibition. This study extends the application scope of the information encounter theory from human health to the field of pet health. It provides empirical evidence and practical references for deepening the application of information encounter theory among pet owners, as well as improving the precision and effectiveness of pet health information services in the mobile media environment.

Based on the above findings, the following recommendations are proposed. (1) For pet owners, it is suggested that they actively improve their information literacy and awareness, cultivate the ability to evaluate the quality of pet health information, transform high-quality incidentally encountered pet health information into effective resources for daily health management, form a sound cycle of using incidentally encountered pet health information, and avoid the absence of internal driving factors. (2) For mobile media platforms, efforts should be made to strengthen the publicity of the Cybersecurity Data Security Management Regulations, improve the quality review mechanism of pet health information, provide authoritative labels for scientifically accurate information, optimize information presentation, and enhance the readability and comprehensibility of content. (3) For operators of animal-related accounts, various forms of pet health promotion can be carried out, such as health video explanations, live interactive sessions, and case interpretation. Differentiated communication strategies can be formulated for different user groups: for users with high information literacy, in-depth professional content can be provided; for users sensitive to information form, visual and practical expression should be emphasized.

In summary, this study reveals the multiple driving paths of pet owners’ usage behavior of incidentally encountered pet health information from a configurational perspective. It extends the application of information encountering theory from human health to pet health. This study provides empirical evidence and practical implications for deepening the application of information encountering theory among pet owners and improving the effectiveness of mobile pet health information services.

## Data Availability

The original contributions presented in the study are publicly available. This data can be found here: https://www.jianguoyun.com/p/DfLWfQ8Q5K3eDRjg5J0GIAA.
